# Genome-wide identification and expression profile of YABBY genes in *Averrhoa carambola*

**DOI:** 10.7717/peerj.12558

**Published:** 2022-01-04

**Authors:** Chengru Li, Na Dong, Liming Shen, Meng Lu, Junwen Zhai, Yamei Zhao, Lei Chen, Zhiting Wan, Zhongjian Liu, Hui Ren, Shasha Wu

**Affiliations:** 1 College of Landscape Architecture, Fujian Agriculture and Forestry University, Fuzhou, Fujian, China; 2 Horticulture Research Institute, Guangxi Academy of Agricultural Sciences, Nanning, Guangxi, China

**Keywords:** *Averrhoa carambola*, YABBY gene family, Genome-wide analysis, Fruit development, RT-qPCR

## Abstract

**Background:**

Members of the plant-specific YABBY gene family are thought to play an important role in the development of leaf, flower, and fruit. The YABBY genes have been characterized and regarded as vital contributors to fruit development in *Arabidopsis thaliana* and tomato, in contrast to that in the important tropical economic fruit star fruit (*Averrhoa carambola*), even though its genome is available.

**Methods:**

In the present study, a total of eight YABBY family genes (named from *AcYABBY1* to *AcYABBY8*) were identified from the genome of star fruit, and their phylogenetic relationships, functional domains and motif compositions, physicochemical properties, chromosome locations, gene structures, protomer elements, collinear analysis, selective pressure, and expression profiles were further analyzed.

**Results:**

Eight *AcYABBY* genes (*AcYABBYs*) were clustered into five clades and were distributed on five chromosomes, and all of them had undergone negative selection. Tandem and fragment duplications rather than WGD contributed to YABBY gene number in the star fruit. Expression profiles of *AcYABBYs* from different organs and developmental stages of fleshy fruit indicated that *AcYABBY4* may play a specific role in regulating fruit size. These results emphasize the need for further studies on the functions of *AcYABBYs* in fruit development.

## Introduction

Rapid radiation of angiosperms to almost all regions of Earth depended largely on the unique trait of producing fruits which protect ovules and seeds during embryo development and ensure seed dispersal at maturation (Ronse De Craene, 2010). Fruits have long been an important energy source for humans due to their important nutritious and medicinal values. Currently, some genes that control fruit development have been studied (Frary et al., 2000; Van der Knaap & Ostergaard, 2017), such as *SUN* (Jiang et al., 2009; Wu et al., 2011; Xiao et al., 2008), *FAS* and *CRC* (YABBY gene family member, Cong, Barrero & Tanksley, 2008; Lee et al., 2005a, 2005b), *Ovate* (Sabatini et al., 2006), *IND* (Dong et al., 2019), and *HECATE* (Schuster, Gaillochet & Lohmann, 2015). Here, we focus on the YABBY gene family.

YABBY genes, which are plant-specific transcription factors, play important roles in the establishment of adaxial–abaxial polarity (Kumaran, Bowman & Sundaresan, 2002), development of lateral organs (Bowman, Smyth & Meyerowitz, 1989), and seed development (Finet et al., 2016). They contain two highly conserved DNA-binding domains: a zinc finger-like domain (C2C2), and helix-loop-helix domain (termed YABBY) (Jang et al., 2004). The six YABBY genes of *Arabidopsis thaliana* can be divided into five groups: FIL/YAB3, CRC, INO, YAB2, and YAB5 (Lee et al., 2005b; Yamada, Ito & Kato, 2004; Yamada et al., 2011). However, the relationship between the five subfamilies remains a mystery due to the lack of a suitable outgroup (Finet et al., 2016). Four of these (*FIL*, *YAB3*, *YAB2*, and *YAB5*) are expressed in both vegetative and reproductive organs and act redundantly to promote lateral organ development. The role of vegetative YABBY genes in core eudicots (Golz et al., 2004) and monocots (Juarez et al., 2004; Juarez, Twigg & Timmermans, 2004) has also been demonstrated. *CRC* and *INO* are regarded as the “floral-specific YABBY genes,” which are restricted to the reproductive organs (Bartholmes, Hidalgo & Gleissberg, 2012; Bowman, 2000; Sarojam et al., 2010; Siegfried et al., 1999). Expression and functional analyses have suggested that *CRC* plays a regulatory role in carpel development in angiosperms (Fourquin et al., 2005; Orashakova et al., 2009; Yamaguchi et al., 2004) and development of nectaries in core eudicots (Lee et al., 2005a). Similarly, *INO* plays an important role in promoting the development of the outer integument (the cell layer surrounding the nucleus) of the ovule to the seed coat (Bowman, 2000; Villanueva et al., 2000).

Given the important role of YABBY genes in plant development, the YABBY gene family has been widely studied in plants, including the dry fruit plants *A. thaliana* (Siegfried et al., 1999), rice (Tanaka et al., 2012), wheat (Zeeshan et al., 2020), *Phalaenopsis* (Chen et al., 2020b), and the fleshy fruit plants tomato (Cong, Barrero & Tanksley, 2008), grape (Zhang et al., 2019), *Punica granatum* (Zhao et al., 2020), and cucumber (Li et al., 2011). However, no such study has been conducted on the star fruit *Averrhoa carambola*, even though two genome drafts have been published (Fan et al., 2020; Wu et al., 2020). The star fruit, also known as carambola, belongs to the family Oxalidaceae, which comprises approximately 780 species in five genera. This plant is remarkable regarding fruit type, fruit morphology, life form, and nyctinastic movement (Shui & Leong, 2006). Therefore, star fruit is an ideal resource for research on fruit development, and genome-wide YABBY gene family analysis will yield novel insights into the key traits that contributed to the diversification of fruits within Oxalidaceae.

## Materials and Methods

### Data sources

A genome sequence (accession: SAMC124704) and a GFF3 file (accession number: GWHABKE00000000) of *Ave. carambola* were downloaded from the China National Center for Bioinformation (https://bigd.big.ac.cn/). The transcriptome data of leaves, inflorescences, bracts, and fruits at different developmental stages were sequenced by Biomark Biotechnology Cooperation, Transcriptome alignment and assembly are performed using Hisat (Kim, Langmead & Salzberg, 2015) and Stringtie2 (Kovaka et al., 2019), respectively. The raw data can be found under the following accession numbers: SRR17036676–SRR17036678, SRR15860439–SRR15860441, SAMN21399627–SAMN21399632.

### Identification and physicochemical properties of YABBY genes in star fruit

To identify all candidate YABBY genes in star fruit, a local BLASTP search with a threshold e-value of 1e^−10^ was performed using *A. thaliana* YABBY protein sequences as query sequences. Identity and coverage areas (>50%) were used as filtering criteria to eliminate inappropriate YABBY genes. In addition, a pfam seed model (PF04690) was obtained from the online database (http://pfam.xfam.org/) and was used for building a hidden Markov model (HMM) file using HMMER3 software with default parameters. The HMM search program was performed to search YABBY genes from .hmm file generated in the previous step. The results of HMM and BLASTP were compared, and repeats were removed manually. Truncated peptides and proteins at the same chromosomal position were eliminated. To further verify the reliability of the selected sequences, the CDD (https://www.ncbi.nlm.nih.gov/) was used for domain analysis to ensure the presence of the YABBY domain in each candidate *AcYABBY* gene. In addition, the protein isoelectric point and molecular weight of *AcYABBY* genes (*AcYABBYs*) were predicted using the ProtParam tool (Gasteiger et al., 1999; https://web.expasy.org/protparam/).

### Analysis of conserved motifs, conserved domains, and subcellular location prediction

To identify shared motifs and structural divergences among the proteins encoded by the YABBY genes, YABBY protein sequences were subjected to analysis using online software MEME 5.1.1’s (Bailey et al., 2006; http://meme-suite.org/tools/meme), and the number of motifs was set to 15. NCBI Batch CD-Search was used to quantitatively predict the structural domains and conserved sites of genes, and DNAMAN v9.0 was used to visualize the domain. Subcellular localizations were predicted using the three protein subcellular location prediction tools Softberry (http://www.softberry.com/), PSORT (https://www.genscript.com/wolf-psort.html?src=leftbar), and LocTree3 (https://www.rostlab.org/services/loctree3/).

### Multiple sequence alignment and phylogenetic analysis

Multiple sequence alignments of YABBY proteins were performed using the MUSCLE alignment function in MEGA 7.0 software with default settings (Kumar et al., 2018). The aligned sequences were saved with a .fasta extension by choosing Export Alignment from the Data menu for exporting the file. After this, the fasta file was converted to a .phy file using EasyCodeML (Gao et al., 2019) and was submitted to CIPRES (https://www.phylo.org/) for phylogenetic analyses (Miller, Pfeiffer & Schwartz, 2010). A Maximum Likelihood (ML) method phylogenetic tree was constructed through RAxML (Stamatakis, 2014) under a GTRGAMMA substitution model with 1,000 bootstraps. The resulting phylogenetic data was exported as a Newick file to produce a phylogenetic tree in the FigTree v1.4.4 (http://tree.bio.ed.ac.uk/software/figtree/), and polar tree layout was chosen to visualize a ring phylogenetic tree.

### Gene structure analysis and chromosomal localization

The GFF3 file of the identified *AcYABBYs* was submitted to the online GSDS v2.0 (http://gsds.gao-lab.org/) (Hu et al., 2014) for gene structure analysis, and the output was saved in SVG format, the convertio, an online image manipulation tool was used to convert SVG format to JPEG format. To determine the chromosomal position of *AcYABBYs*, the sequences of *AcYABBYs* were mapped to the genome of star fruit, according to the coordinates of each YABBY gene on the genome.

### Prediction of secondary structures of *AcYABBYs*

To investigate the secondary structure of *AcYABBYs*, the whole AcYABBY protein was submitted and predicted using http://bioinf.cs.ucl.ac.uk/.

### Prediction of promoter elements

To identify putative *cis*-acting elements in the promoter, we used TBtools to obtain 2,000 bp gene sequence upstream of the promoter codon from the star fruit genome sequence (Chen et al., 2020a). *Cis*-acting elements in the promoter region were analyzed using PlantCARE (Higo et al., 1999). Microsoft Excel 2010 was applied to the screen promoter elements to produce a histogram.

### Colinear and selective pressure

To identify the pattern of gene duplication, MCscanX (Wang et al., 2012) was used to analyze YABBY genes in star fruit vs *A. thaliana*, and star fruit vs grape. Two piars of blast result files from star fruit vs *A. thaliana*, *A. thaliana* vs star fruit and star fruit vs grape, grape vs star fruit were merged. MCscanX was then used to examine the merged blast file and the merged gff3 file that met the specifications. The dual_synteny_plotter script program was used to visualize the merged gff3 file, the collinearity file obtained in the previous step, and the ctl file. To assess the selection pressure of genes encoding YABBY proteins, the ratio of Ka/Ks (an indicator of selective pressure) was used to evaluate its evolutionary pressure. MAFFT 7.0 (Nakamura et al., 2018) was applied to align the protein sequence of YABBY genes in star fruit, and then the pal2nal.pl and csplit scripts were used to compare the aligned protein sequence with the CDS sequences of *AcYABBYs* and to split the aligned file. The split files were then used to form new gene pair files, and the parseFastaIntoAXT.pl script was used to convert the fasta file into an axt file. The KaKs_Calculator software (Wang et al., 2009) was used to calculate Ka/Ks values of the gene pairs.

### Expression analysis of *AcYABBYs*

To obtain more information regarding the roles of YABBY genes in star fruit, RNA sequencing data were used to quantify the expression levels of YABBY genes in different organs and developmental stages from star fruit, and the raw count number of mapped reads for each *AcYABBY* (reads per kilo base per million mapped reads) was calculated using StringTie (Kovaka et al., 2019). A gene expression profile heatmap was then produced using Tbtools (Chen et al., 2020a).

### RNA extraction and Reverse Transcription qPCR (RT-qPCR)

Since the ripening cycle of star fruit is about 2 months, we use the fruits 20, 40, 60 days after pollination (F-DAP20, F-DAP40, F-DAP60) to represent the early, middle and late stages of fruit development. Leaves, inflorescences, buds, fruits from F-DAP20, F-DAP40, F-DAP60 were collected and frozen in liquid nitrogen for 30 min, and were stored at −80 °C until RNA extraction which was performed as quickly as possible (Fig. S1). Total RNA was extracted using the RNAsimple Plant Kit (Tiangen Biochemical Technology Company, Beijing, China) following the manufacturer’s instructions.

RT-qPCR was performed to further confirm the reliability of the expression profile results using all genes (from *AcYABBY1* to *AcYABBY8*). Total RNA of all collected samples was extracted using the TIANGEN DP441 Reagent (TIANGEN, Beijing, China) following the manufacturer’s instructions. RT-qPCR analysis was performed using a Roche detection system (Roche, Switzerland) with SYBR Green assays. Gene-specific primers for the RT-qPCR analysis of eight selected genes and a reference gene are listed in Table S1. The reaction conditions were 4 min at 95 °C and 40 cycles of 10 s at 95 °C and 40 s at 60 °C. The specificity of the amplicon for each primer pair was verified by melting curve analysis. The melting curve conditions were 95 °C for 15 s, 60 °C for 1 min, 95 °C for 30 s, 60 °C for 15 s, and each sample showed only one melting temperature peak. All experiments were performed in three biological replicates, and each replicate was measured thrice. The log2 fold change was calculated using the 2−ΔΔCT method with Yangtao2017018, a homolog of beta-actin (ACTB), as a reference gene to normalize the target gene expression and to correct for variation between samples.

## Results

### Identification and physicochemical properties of YABBY genes in star fruit

Local BLAST and HMM analyses were performed, and genes such as pseudogenes, premature stop codons genes, or those without a complete YABBY domain were removed. Finally, eight remaining putative functional YABBY genes with high confidence were identified in *Ave. carambola*. All genes contained the YABBY domain (Fig. S2). In addition, the YABBY genes varied substantially in the length of encoded protein sequences, ranging from 169 to 235 bp (Table S2). The molecular weight of these *AcYABBYs* ranged from 18,053.34 to 25,197.26 Da. Most of the *AcYABBYs* exhibited alkaline isoelectric points higher than 8.00, with the highest being 9.56 for *AcYABBY7*, while two proteins had acidic isoelectric points below 8.00, of which *AcYABBY2* had the lowest at 5.22.

### Analysis of conserved motifs and subcellular location predictions

To identify motifs in the *AcYABBYs*, motifs of YABBY proteins in *A. thaliana* and star fruit were analyzed using the online analysis tool MEME, and 15 motifs were set. Motif 1 and motif 2 encoded the YABBY and zinc finger domains, respectively, which are the most conserved domains with 50 and 48 amino acids, respectively (Fig. S3). All YABBY genes in *A. thaliana* and star fruit had these two motifs. Motif 3, motif 5, and motif 8 were specifically distributed in the FIL/YAB3 subclade of *A. thaliana* and star fruit, but motif 6, motif 11 were specifically distributed in the FIL/YAB3 subclade of *A. thaliana* or star fruit, respectively (Fig. S3). Motif 4 and motif 7 were present in the YAB2 and YAB5 subclades. Motif 9 and motif 13 were specifically distributed in the INO clade. Motif 10 and motif 14 were specifically distributed in the YAB2 clade. The motif 12, motif 15 were specifically distributed in the YAB5, CRC subclade, respectively. Notably, though *AcYABBY3*, *AcYABBY4*, and *YAB2* belonged to YAB2 clade, only *ACYABBY3* had the same motif structure as *YAB2*, and *ACYABBY4* has no other motifs except the common motif 1 and motif 2 of eight YABBY genes (Fig. S3). In addition, the results from subcellular location predictions showed that all YABBY proteins were most likely to be located in the nucleus, suggesting that they function on the nucleus or directly on the nucleus.

### Classification and phylogenetic analyses of *AcYABBYs*

**Figure 1:**
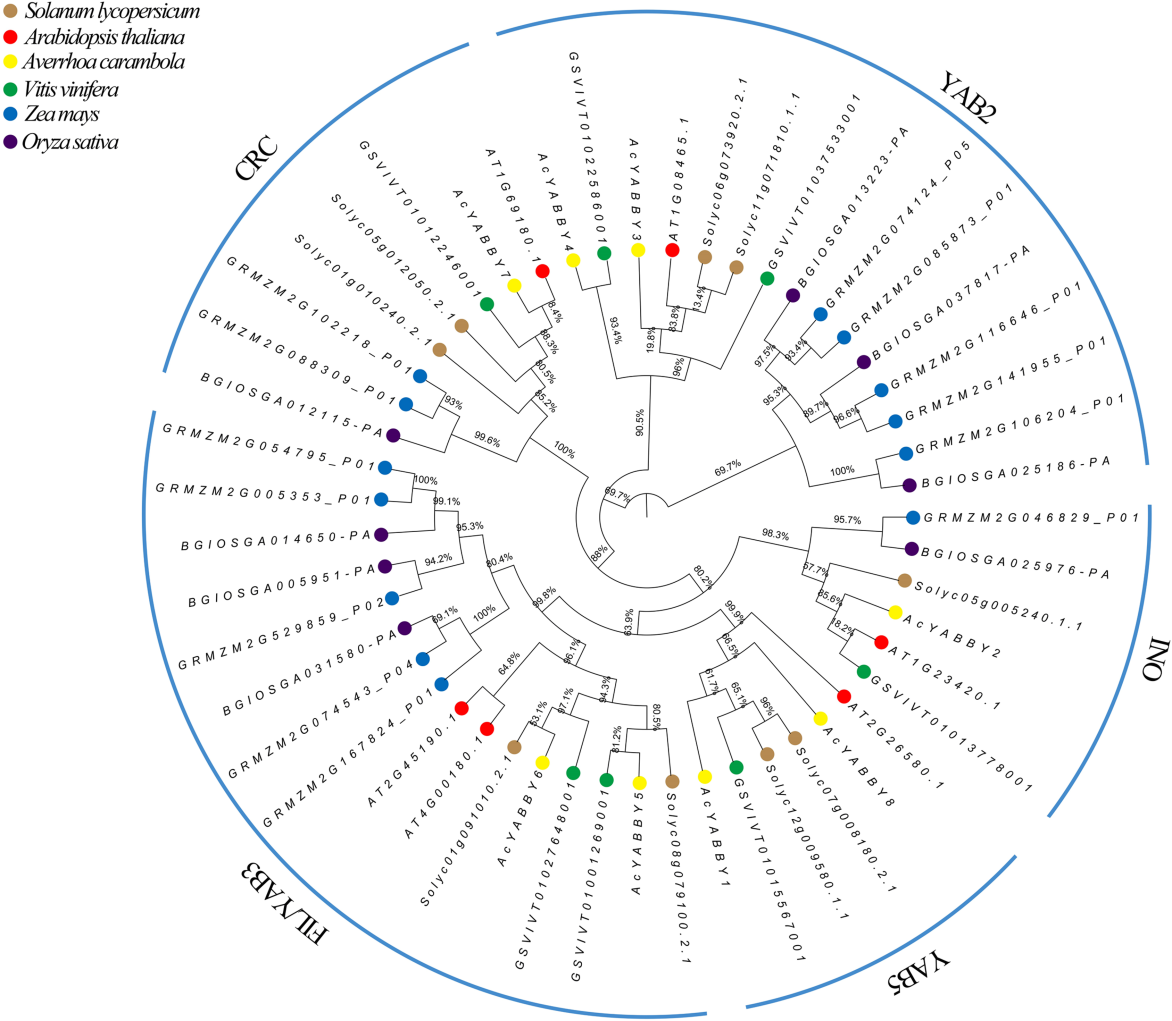
Phylogenetic analysis of *Ave. carambola*, *Z. mays*, *O. sativa*, *V. vinifera*, *S. lycopersicum*, and *A. thaliana*.

The phylogenetic analysis results showed that star fruit *AcYABBYs* can be divided into five subclades, namely, FIL/YAB3 (two members, *AcYABBY5* and *AcYABBY6*), YAB2 (two members, *AcYABBY3* and *AcYABBY4*), YAB5 (two members, *AcYABBY1* and *AcYABBY8*), INO (one member, *AcYABBY2*), and CRC (one member, *AcYABBY7*), and the total number of *AcYABBYs* is far below that in *Z. mays* (Table S4, Fig. 1). Specifically, the two monocot plants rice and maize have more *YAB3-*like genes (three and five members, respectively) than the other examined dicotyledons, in which *A. thaliana*, tomato, grape, and star fruit have six, nine, seven and eight members, respectively (Table S4). Similar to the other two tested species with fleshy fruit, star fruit had two *YAB2-*like members, which is less than that in maize (five members) and rice (three members) but more than that in *A. thaliana* (only one member). Interestingly, the *YAB5-*like genes were absent in the monocot plants rice and maize but present in other plants, and their number seemed to be conservative (one or two). Similarly, the FIL/YAB3 subclade consisted of two parts: monocots and dicots. Among all the tested plants, both tomato and maize have two *CRC-*like genes, there was only one *CRC-*like gene present in the other plants, and only one *INO-*like gene was found in all examined species.

### Structure and chromosome distribution of YABBY genes in star fruit

A total of eight *AcYABBYs* in star fruit were distributed on five chromosomes (Fig. S4). Chr.02, Chr.06, and Chr.08 contained equal numbers of *AcYABBY* (only one member), whereas Chr.04 contained two *AcYABBYs* and Chr.09 contained three *AcYABBYs* members. In addition, a pair of homologous genes, *AcYABBY1* and *AcYABBY8*, was located on the same chromosome, but two other homologous gene pairs (*AcYABBY3* and *AcYABBY4*, *AcYABBY5* and *AcYABBY6*) were located on different chromosomes. Two floral-specific genes (*INO*-like gene *AcYABBY2* and *CRC*-like gene *AcYABBY7*) were located on the same chromosome.

The results suggested that all of the *AcYABBYs* have introns and exons. *YAB2-*like (*AcYABBY3* and *AcYABBY4*) and *CRC-*like (*AcYABBY7*) *AcYABBY*s had fewer introns than the other *AcYABBYs*, while the lengths of introns in the *YAB2-*like (*AcYABBY3* and *AcYABBY4*) *AcYABBY*s were greater than those in other *AcYABBYs*, at over 2 kb (Fig. S5). Among the eight *AcYABBYs*, there were six exons in *YAB2-*like (*AcYABBY3* and *AcYABBY4*), *CRC-*like (*AcYABBY7*), and one *YAB1-*like member *AcYABBY8*, compared to the seven exons in other *AcYABBYs* (Fig. S5). In addition, regardless of whether it was *YAB1-*like, *YAB2-*like, or *YAB5-*like, the gene structure showed a high degree of similarity in spite of slight differences existing in the introns and exons, which may be because of their phylogenetic relationship. *AcYABBYs* contained highly conserved gene structures, especially regarding orthologous genes (Fig. S5).

### Secondary structure analysis of YABBY proteins

The secondary structure analysis showed that all the star fruit AcYABBY proteins contained α-helix, strand, and coil structures (Fig. S6). Homologous genes usually have generally similar secondary structures and stable secondary structure types at nearly the same position as the base pair (bp). For example, *AcYABBY3* and *AcYABBY4* had a helix and a strand between 117 bp and 123 bp, and between 12 bp and 15 bp, respectively.

### *Cis*-acting elements located in YABBY gene promoters

To explore the possible regulatory functions of *AcYABBYs*, the 2,000 bp promoter regions of eight *AcYABBYs* were submitted to the PlantCARE database for the identification of putative *cis*-elements. A total of 14 types of elements were identified: the zein metabolism regulation element, light responsiveness element, MeJA-responsiveness element, anaerobic induction element, abscisic acid responsiveness element, salicylic acid responsiveness element, meristem expression element, seed-specific regulation element, circadian control element, low-temperature responsiveness element, auxin-responsive element, endosperm expression element, defense and stress responsiveness, and wound-responsive element (Fig. S7). All *AcYABBYs* contained multiple promoters and all contained light responsiveness element, while most subfamilies obtained salicylic acid responsiveness element, except for the YAB3 subfamily (Fig. S7). Most of the *AcYABBYs* contained more than five elements, the smallest was *AcYABBY5* with three and the largest was *AcYABBY6* with nine elements (Fig. S7). Interestingly, the abscisic acid responsiveness element, and the anaerobic induction element were absent in *AcYABBY5* and *AcYABBY3*, respectively, but their homologous genes and other *AcYABBYs* all contained these elements (Fig. S7). In addition, some promoter elements were specific to a single *AcYABBY*, including wound-responsive element specific to *AcYABBY7*, endosperm expression element specific to *AcYABBY6*, and low-temperature responsiveness element specific to *AcYABBY6* (Fig. S7). Moreover, some elements were specific to two *AcYABBYs*, for example, the seed-specific regulation element specific to *AcYABBY4* and *AcYABBY6*, the auxin-responsive element specific to *AcYABBY1* and *AcYABBY6*, the circadian control element specific to *AcYABBY3* and *AcYABBY4*, and the zein metabolism regulation element specific to *AcYABBY3* and *AcYABBY8* (Fig. S7).

### Synteny and evolutionary analysis of *AcYABBYs*

**Figure 2:**
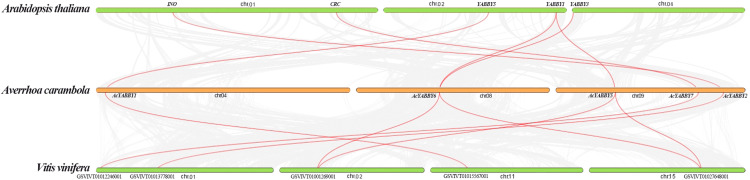
The collinear correlation for all the YABBY genes is displayed between *A. thaliana* and star fruit, and grape and star fruit.

We investigated collinear relationships among the orthologous YABBY genes from star fruit, grape, and *A. thaliana* to find putative evolutionary events. There are six, seven, and eight YABBY genes in *A. thaliana*, grape, and star fruit, respectively. The results showed that all of YABBY genes showed one-to-one corresponding relationship in *A. thaliana* - star fruit, and star fruit-grape (Fig. 2).

In the process of evolution, genes usually face various selection pressures, such as positive selection (Ka/Ks > 1), neutral selection (Ka/Ks = 1) and purifying selection (Ka/Ks < 1), understanding the selection pressure of genes plays an important role in studying the evolution of genes (Khan et al., 2019). To explore evolutionary constraints among the homologous *AcYABBYs*, we calculated Ka, Ks, and the ratio for Ka/Ks. In our study, 28 gene pairs were identified using KaKs_Calculator. Ka, Ks, and Ka/Ks values between each pair are listed in Table S5. The Ka of *AcYABBYs* ranged from 0.14116 to 0.591704, the Ks from 0.877205 to 3.51706, and the Ka/Ks from 0.104914 to 0.313231, suggesting that all of them had undergone strong purifying selection (Table S5).

### Expression patterns of YABBY genes in star fruit

**Figure 3:**
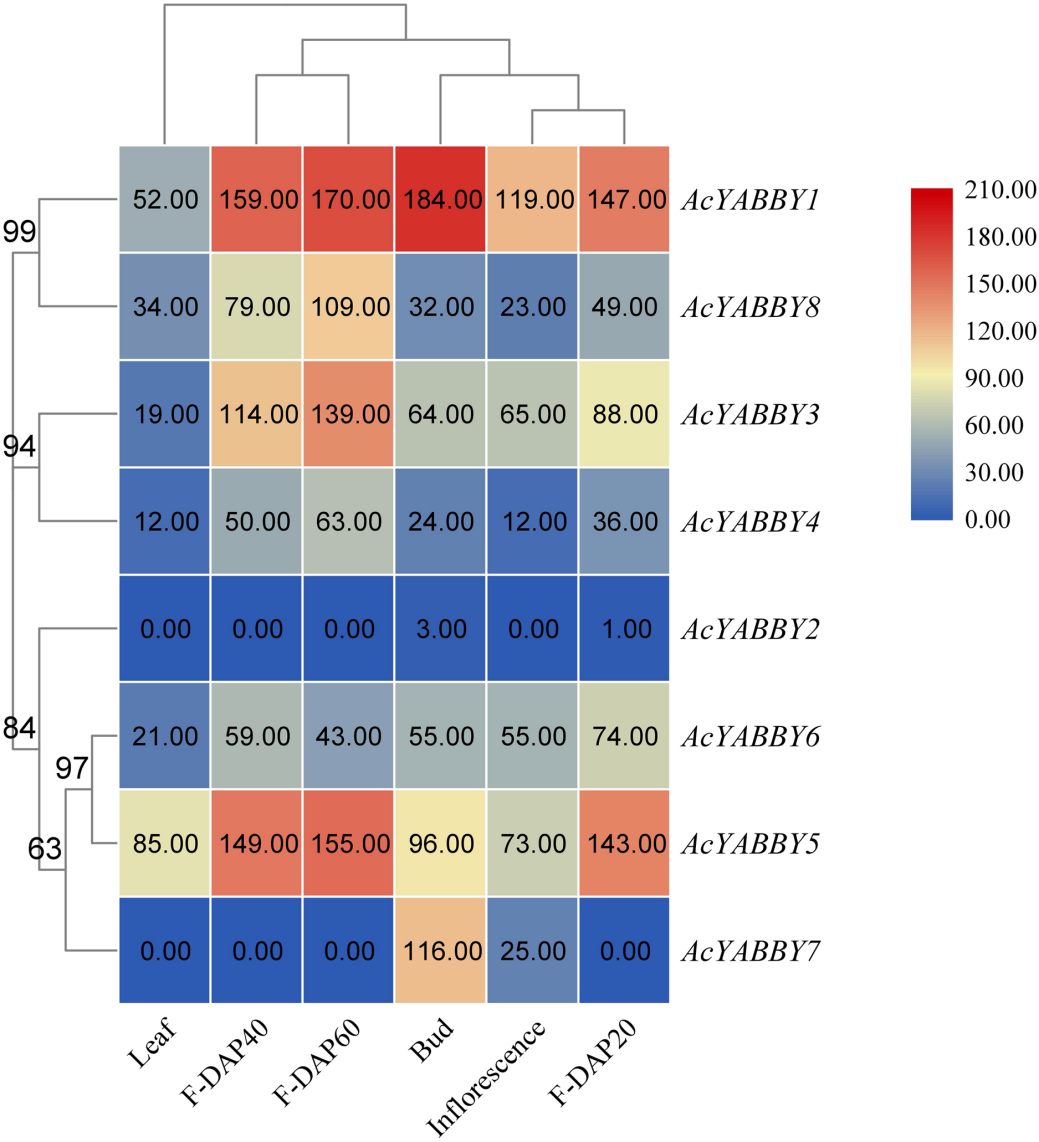
Heatmap of expression profiles for *AcYABBYs* in different tissue and stages. Note: The FPKM values were labelled on boxes, the fruits 20, 40, 60 days after pollination (F-DAP20, F-DAP40, F-DAP60) represent the early, middle and late stages of fruit development, respectively.

To further elucidate the function of the star fruit YABBY gene family, eight *AcYABBYs* were used to produce an expression profile heat map using Tbtools, and three biological replications were performed for each tissue to obtain accurate expression levels. *AcYABBY2* expression was extremely low and almost undetectable in all tissues (Fig. 3). *AcYABBY7* was at an extremely low expression level in the leaf and fruit at different developmental stages but exhibited a relatively high expression level in the inflorescences, bud (Fig. 3). Overall, expression levels of *YAB2-*like, *YAB5-*like, and *FIL-*like genes in reproductive organs were slightly higher than those in the leaf, and F-DAP40 expression levels of nearly all *AcYABBYs* expect for *AcYABBY6* and *AcYABBY7* were either lowest or highest during fruit development (Fig. 3).

**Figure 4:**
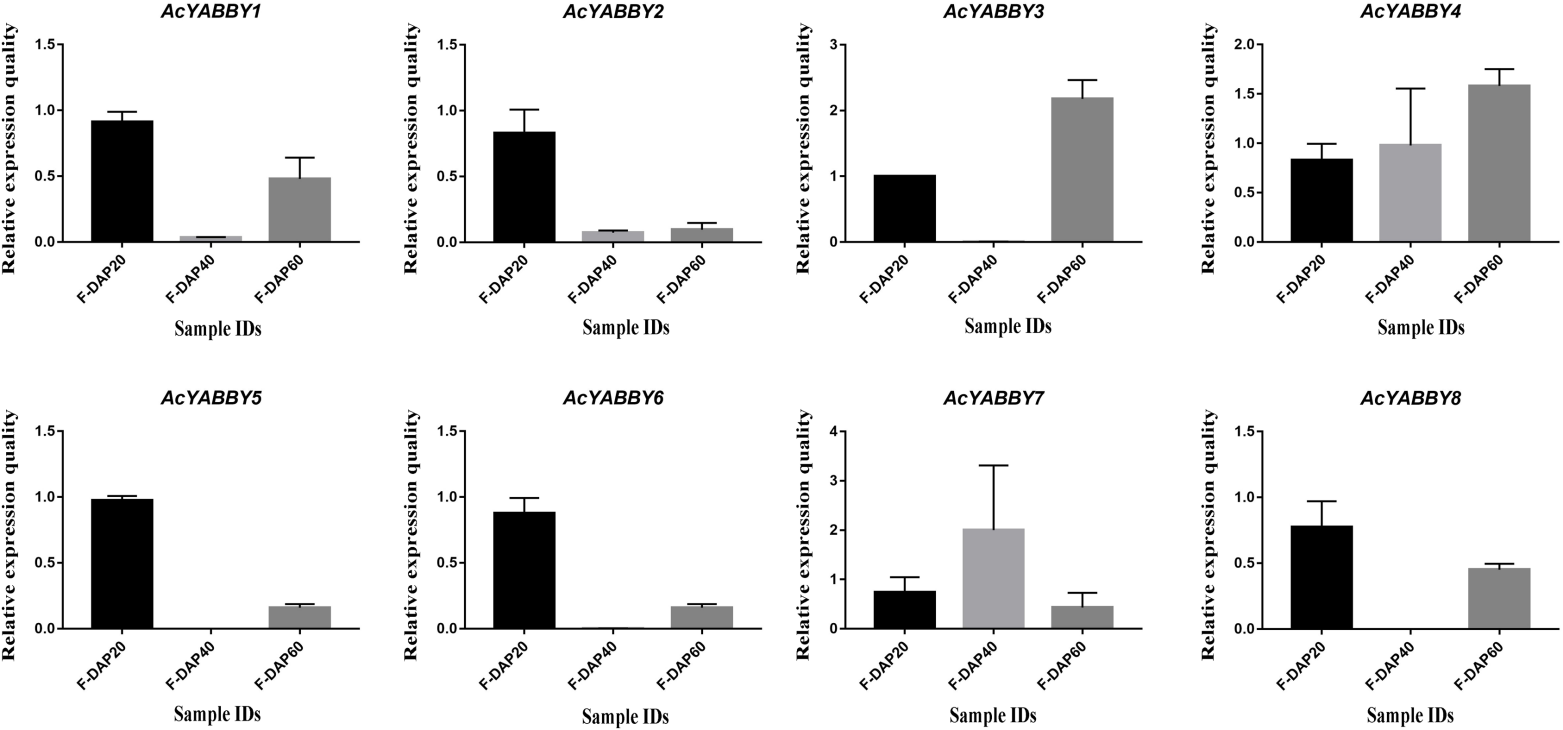
Relative expression histogram for *AcYABBYs* in different tissue and stages. The color scale above represents different stages of fruit. Note: Black, Light brown, and Dark brown indicated F-DAP20, F-DAP40, F-DAP60, respectively.

To further validate the accuracy of transcriptome sequencing, all *AcYABBYs* in the star fruit during the developmental stages of fruit were subjected to RT-qPCR. The expression levels of *AcYABBYs* can be divided into four categories based on the trend of relative expression (Fig. 4). *AcYABBY1*, *AcYABBY2*, *AcYABBY5*, *AcYABBY6*, and *AcYABBY8* showed a trend of an initial decrease followed by an increase, and the expression level of F-DAP20 was highest. *AcYABBY7* showed a diametrically opposite trend. Expression levels of *AcYABBY4* gradually increased during fruit development, *AcYABBY3* also showed a trend of an initial decrease followed by an increase, and the expression levels of F-DAP60 was highest (Fig. 4).

## Discussion

TFs play an important role in manifold biological processes by controlling the expression of target genes (Riechmann et al., 2001; Sharma, Bhalla & Singh, 2013). YABBY gene family encodes TFs that are involved in the development of the leaf, flower, and fruit (Bartholmes, Hidalgo & Gleissberg, 2012). Regarding basal angiosperms, five YABBY genes have been described in *Nymphaea colorata* and *Amborella trichopoda* (Yamada et al., 2011). In the model plant *A. thaliana*, six YABBY genes have been identified (Bowman, Smyth & Meyerowitz, 1989), eight members in the representative monocot plant rice (Toriba et al., 2007), and nine YABBY genes in tomato as an economically important crop (Huang et al., 2013). In this study, a total of eight *AcYABBYs* were identified in star fruit, and all subclades were present in star fruit.

Although the physicochemical properties of most YABBY proteins are conserved, there are two proteins that are significantly different from the others, which seemed to indicate that *AcYABBYs* could play different roles in alternate microenvironments. In addition, all *AcYABBYs* are located in the nucleus, suggesting that they might play transcriptional regulatory roles directly in the nucleus.

An accurate understanding of the evolutionary history of genes is essential for inferring changes in gene functions and developmental modules (Theißen, 2005; Thornton & DeSalle, 2000). A total of 51 YABBY protein sequences were used to construct a phylogenic tree. Consistent with previous research, eight *AcYABBYs* can be divided into five subclades (YAB3/FIL, YAB2, YAB5, CRC, and INO). In general, the distribution of *AcYABBYs* in star fruit is consistent with the distribution of YABBY family in most plants, indicating that the plant-specific YABBY gene family is evolutionarily conserved. However, there are more YABBYs in *Z. mays* than in other species (Tanaka et al., 2012), especially the FIL/YAB3 and YAB2 subclades, which can be interpreted as small-scale gene duplication. It is worth noting that monocot species, including rice and maize, also do not have *YAB5*-like genes, whereas their homologous *YAB2*-like genes are present in monocots and dicots. *FIL*-like genes, which are homologs to *YAB2*-like genes, also distribute in monocots and dicots. The separation of *YAB2* and *YAB5* occurred after the divergence of monocots and dicots, and *YAB5* was subsequently lost in monocots (Chen et al., 2020b; Almeida et al., 2014). Our results are, thus, in line with those of previous studies.

Gene structure is typically conserved in the evolution process (Fedorov, Merican & Gilbert, 2003; Rogozin et al., 2003). It was found that the gene structures of the star fruit *AcYABBYs* are the same as those of YABBYs in *A. thaliana*, in which the *FIL*/*YAB3-*like has more exons and the *YAB2-*like gene has more introns. These results indicated that the YABBY gene family was conserved during evolution. In general, the *FIL*/*YAB3-*like, *YAB2-*like, and *YAB5-*like genes in star fruit have higher numbers of introns than *CRC-*like and *INO-*like genes, suggesting that the former three genes are more conserved than the latter two. It has been shown that the density of splicing regulatory sequences increased with increasing intron length (<1.5 kb), while increasing intron lengths (>1.5 kb) are associated with increased splicing site strength (Dewey, Rogozin & Koonin, 2006). Therefore, various intron patterns between genes may present evolutionary conservation of expression or splicing regulation in star fruit. In addition, evolutionally conserved genes have a larger intron burden and present a positive correlation between the level of evolutionary conservation and the intron region size of eukaryotic genes (Gorlova et al., 2014). Similarly, the *FIL*/*YAB3-*like, *YAB2-*like, and *YAB5-*like genes have a wider expression range and might participate in more important biological functions than *CRC-*like and *INO-*like genes in star fruit, which is consistent with previous studies on *A. thaliana* (Siegfried et al., 1999).

*Cis* elements in the promoter region play important roles in the regulation of gene expression, and presence or absence of these elements affect gene expression (Bilas et al., 2016). Finding the conserved cis-acting motifs can be used to predict the function and potential interactions of genes (Heidari et al., 2019). Previous studies revealed that YABBY genes are involved in important biological processes (Bowman, 2000; Siegfried et al., 1999). To better understand the regulation roles of *AcYABBYs*, the *cis* elements in their promoter regions were investigated. Many types of plant regulatory elements including stress-responsive, hormone-related, reproductive-related, circadian control, and other regulatory elements were identified in the putative promoters of *AcYABBYs*. MeJA and ABA not only regulate plant growth and development, but are also associated with plant defenses against wound, disease, osmotic challenges, and other adverse environmental factors (Anderson et al., 2004; Ellis & Turner, 2001; Yuan & Zhang, 2015; Zhang et al., 2020). In addition, ABA and JA are involved in a wide range of fruit development processes, including fruit ripening onset, ripening process and fruit quality trait (Yuan, Li & Leng, 2020). They can promote the ripening of different fruits by up-regulating the expression of ethylene biosynthesis genes, such as studies in plums, tomatoes and etc. (Li, Chen & Grierson, 2021; Martínez-Esplá et al., 2014; Zhang, Bing & Ping, 2009). In present study, ABA responsiveness element and JA -responsiveness element widespread in most of *AcYABBYs*, suggesting *AcYABBYs* may participate in fruit development. Previous studies showed that YABBY genes can respond to various stresses and participate in ovule development (Yang et al., 2018). In addition, several auxin-responsive proteins play important roles in floral organ growth in *A. thaliana* (Ghelli et al., 2018). In our study, *AcYABBY1* and *AcYABBY6* were found to possess an auxin-responsive element. In the study in tomatoes and pears, salicylic acid was able to show many aspects of the fruit, such as quality, yield and ripeness (Chakma et al., 2021; Changwal et al., 2021; Shi et al., 2021). *AcYABBY2* and *AcYABBY4* contained the most salicylic acid responsiveness elements in the current study, which seems to mean that they may play an important role in the development of fruits. Previous studies showed that YABBY genes can regulate flower and inflorescence development in rice and *P. granatum* (Tanaka et al., 2012; Zhao et al., 2020). Like the YABBY gene in *A. thaliana*, we speculated that *AcYABBYs* exert similar functions. Such a wide range of *cis*-acting elements is consistent with the important roles of YABBY genes in plant growth and development. Moreover, YABBY genes were also found to participate in leaf development in previous studies, and in the current study, *AcYABBY5* and *AcYABBY1* presented relatively high expression levels, which may imply that they are involved in leaf development. Previous studies showed that YABBY genes play essential roles in seed and fruit (Finet et al., 2016). In the present study, the seed-specific regulation element and the endosperm expression element were present in *AcYABBY4* and *AcYABBY6*, implying that they may have similar functions, though the heatmap did not support this hypothesis; however, their homologous genes presented differential expression between vegetative organs and reproductive organs in star fruit. Additionally, the circadian control element was present in *YAB2*-like genes *AcYABBY3* and *AcYABBY4*. Previous studies showed that the nyctinastic movement of *Oxalis* is closely related to circadian rhythm (Tanaka et al., 1989), and we also observed that the nyctinastic movement of *Averrhoa*, a sister genus of *Oxalis*, is also primarily related to circadian rhythm (unpublished data), we therefore that the YABBY gene may be involved in this interesting movement, which remains to be investigated. Such a wide range of *cis*-acting elements is consistent with the multiple roles of YABBY genes involved in plant growth, development, reproduction, and stress.

Gene duplication is an important force which plays a vital role in the process of genome evolution and functional divergence (Moore & Purugganan, 2003). Dispersed, singleton, WGD (whole genome duplication), or segmental and tandem duplications contributed to the patterns of duplications (Freeling, 2009). Generally speaking, tandem duplication and fragment duplication are the two predominant processes in the evolution of gene families (Cannon et al., 2004). It is reported that the WRKY family is likely to increase by tandem duplication and fragment duplication (Guo et al., 2014). Collinearity analysis showed that all the genes were in a one-to-one relationship, indicating that WGD did not occur in the star fruit. Paralogous genes distributed on different chromosomes are usually considered fragment duplications, and those genes on a single chromosome are considered to be tandem repeat genes (Cai et al., 2013). Gene duplication results have principal roles in the evolutionary expansion of gene family members in plants, which promotes adaptation to diverse environmental conditions (Faraji et al., 2020). In the current study, the six paralogous YABBY genes in star fruit were located on the same chromosome or on different chromosomes, indicating that tandem and fragment duplications contributed to the expansion of YABBY genes in star fruit.

Here, we present a comprehensive investigation of *AcYABBYs* expression levels in different tissues and at three fruit developmental stages in star fruit. Based on the expression profiles, it has been found that the expression levels of *AcYABBYs* are different from those in other plants. For example, both *CRC* and *INO* were expressed only in the reproductive development stages of *A. thaliana*, *Bienertia rapa*, and *B. sinuspersici*, indicating their conserved functions in carpel morphogenesis, pistil differentiation, floral meristem, and ectoderm development (Soundararajan et al., 2019; Yamada et al., 2011), In *P. granatum*, *PgCRC* showed higher expression levels in leaves, hermaphrodite flowers, functional stamens, and skins, especially in leaves and hermaphrodite flowers, but the other flower-specific gene *PgINO* showed higher expression levels only in hermaphrodite flowers and in the exocarp and almost no expression in the root and pericarp (Zhao et al., 2020). However, in the present study, they presented extremely low or even undetectable expression. In addition, previous studies showed that *FIL*, *YAB2*, and *YAB3* are related to abaxial domains of leaf-derived organs such as cotyledons, leaves, and flower organs (Sarojam et al., 2010; *Sawa et al., 1999a;* 1999b). *FIL/YAB3-*like YABBY genes in rice have been shown to be involved in the maintenance of meristem functions (Tanaka et al., 2012; Tanaka, Toriba & Hirano, 2017). *OsYAB4*, defined as FIL/YAB3 subclade, may also be involved in the vascular system of rice as it dominates the phloem tissues (Liu et al., 2007). *AcYABBY5* and *AcYABBY6*, homologous genes to *FIL* in star fruit also present no-specific expression. In tomato, a *YAB2-*like gene, *FAS*, was critical for fruit size and shape (Cong, Barrero & Tanksley, 2008). Consistent with *FAS*, expression of *YAB2*-like genes in star fruit in reproductive organs was higher than that in vegetative organs. *AcYABBY1*, a *YAB5-*like gene, is highly expressed in flowers, fruits of different developmental stages than that in leaves, indicating that it may be involved in fruit development in star fruit. However, the RT-qPCR results are slightly different from the transcriptome sequencing results, which may be explained by the imperfect correlation between sequencing samples and RT-qPCR samples, thus given the circumstances, we rely on the RT-qPCR results. *AcYABBY4* expression was supported by transcriptome data and RT-qPCR data, speculating that it is a key gene involved in fruit size development. Based on these results, we speculate that the different expression patterns of YABBY genes in star fruit and *A. thaliana* may account for the sub-functionalization and neofunctionalization of YABBY genes.

## Conclusion

In summary, we first systematically performed genome-wide bioinformatic analyses of *AcYABBYs* in star fruit. Eight YABBY genes were identified and divided into five subfamilies. The genes in same subclade have similar conserved motifs and structure, but chromosome distribution seems to have no correlation with phylogenetic relationship. Selective pressure analysis suggested that all *AcYABBYs* had undergone strong purity selection, which implies that the YABBY gene family is highly conserved. In addition, expression profiling of *AcYABBYs* in various organs, especially fruit at different developmental stages, was performed. RT-qPCR analysis was performed to verify the transcriptome data. Taken together, the RNA-sequencing and RT-qPCR results showed that a *YAB2*-like gene, *AcYABBY4*, may play the same role as the *YAB2*-like gene *FAS* in tomatoes, which regulates fruit size and development. Our results lay a foundation for the further understanding of the functional characteristics of the YABBY gene family in star fruit and provide a better understanding of the structure–function relationship among the members of the YABBY gene family. Additionally, our study provides comprehensive information and novel insights into the functions of YABBY genes in plants. Further studies may help understand the molecular basis of many important traits such as flower and fruit development and other physiological processes.

## Supplemental Information

10.7717/peerj.12558/supp-1 Supplemental Information 1 Sample preparations. A: flower bracts; B: inflorescences; C and D: anatomical and front views of the fruits, respectively. (Scale bar: C and D, 2 cm).

10.7717/peerj.12558/supp-2 Supplemental Information 2 Domain of YABBY genes in star fruit.

10.7717/peerj.12558/supp-3 Supplemental Information 3 Conserved motifs of YABBY genes in *A. thaliana* and star fruit were predicted by MEME. Grey lines represent the non-conserved sequences, and four conserved motifs are indicated by different colors with numbered boxes.

10.7717/peerj.12558/supp-4 Supplemental Information 4 Chromosomal locations of *AcYABBYs*. A total of five chromosomes of star fruit were labeled with their names, Chr.02, Chr.04, Chr.06, Chr.08, and Chr.09, which are indicated at the top of each bar. The position of *AcYABBYs* on the chromosome was drawn by online software MG2C (http://mg2c.iask.in/mg2c_v2.0/) based on GFF file.

10.7717/peerj.12558/supp-5 Supplemental Information 5 Gene structure of the *AcYABBYs* in star fruit. Exons and introns are represented by yellow rectangle and black lines, respectively. The lengths of exons and introns for each *AcYABBY* gene are shown proportionally.

10.7717/peerj.12558/supp-6 Supplemental Information 6 The analysis of YABBY protein secondary structures. Different color blocks represent different secondary structures.

10.7717/peerj.12558/supp-7 Supplemental Information 7 Cis-acting elements of *AcYABBYs* in promoter regions. *Note:* The numbers of different cis-elements are presented in the form of bar graphs and similar cis-elements are exhibited with the same colors.

10.7717/peerj.12558/supp-8 Supplemental Information 8 The identified YABBY proteins in star fruit.

10.7717/peerj.12558/supp-9 Supplemental Information 9 The sequences used for phylogenetic analysis.

10.7717/peerj.12558/supp-10 Supplemental Information 10 Supplementary materials.

## Additional Information and Declarations

### DNA Deposition

The following information was supplied regarding the deposition of DNA sequences: The RNA-seq raw data of fruits from three developmental stages are available at GenBank: SRR17036678, SRR17036677, SRR17036676. The RNA-seq raw data of leaf, bract, inflorescence are available at Genbank, respectively: SRR15860439, SRR15860440, SRR15860441. Sequences are available at GenBank: BankIt2384345 Yangtao010607 MW014332 BankIt2384345 Yangtao021545 MW014333 BankIt2384345 Yangtao006609 MW014334 BankIt2384345 Yangtao003754 MW014335 BankIt2384345 Yangtao012644 MW014336 BankIt2384345 Yangtao002777 MW014337 BankIt2384345 Yangtao017952 MW014338 BankIt2384345 Yangtao005911 MW014339

### Data Availability

The following information was supplied regarding data availability: The YABBY protein sequences and other data used are available in the Supplemental Files.
