# Supplementary figures and images for: Genome-wide identification and expression profile of YABBY genes in Averrhoa carambola
Source: PeerJ. 2022 Jan 4;10:e12558. doi: 10.7717/peerj.12558 (PMC8740515; doi:10.7717/peerj.12558)

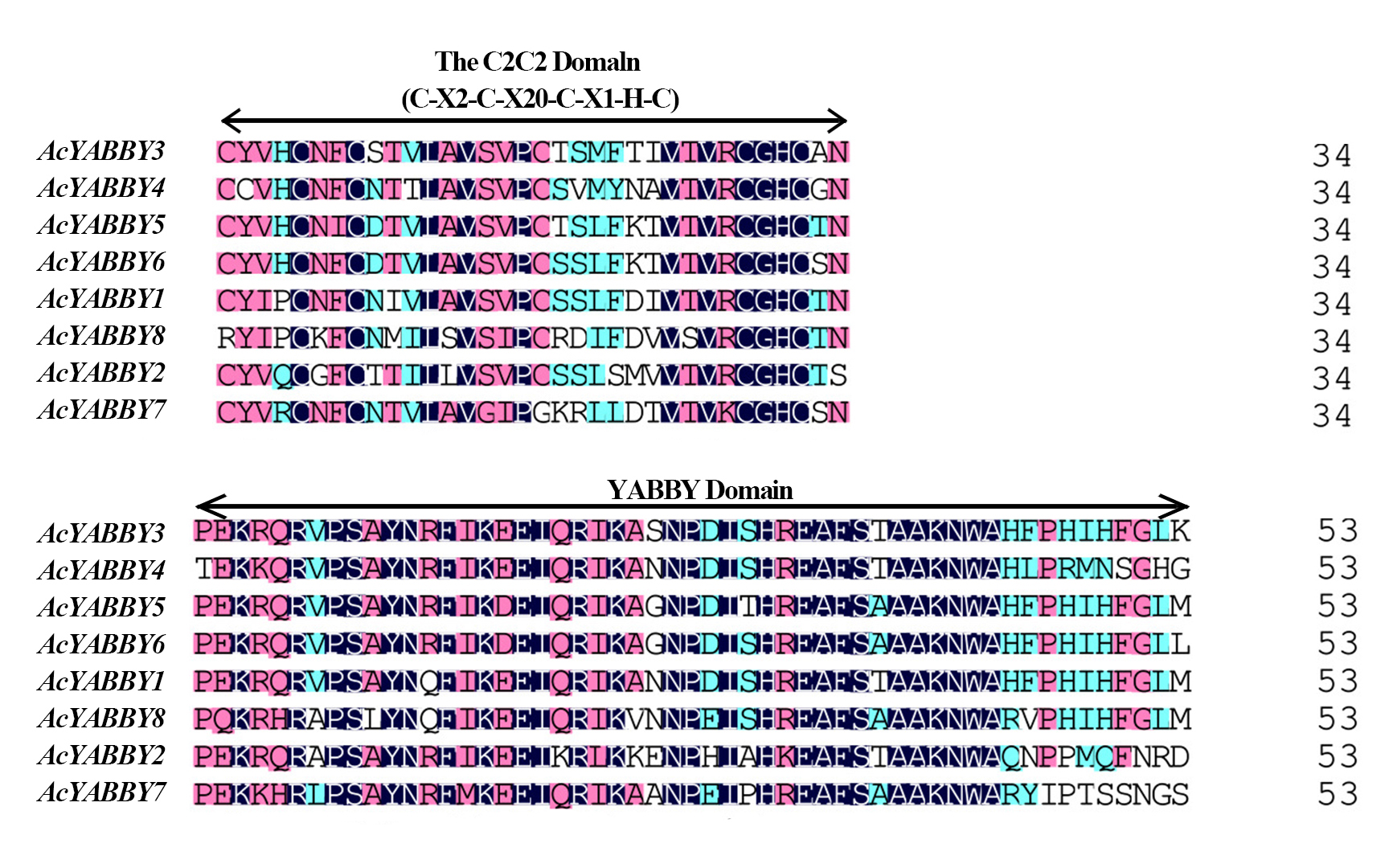

Supplement: Supplemental Information 2 [file peerj-10-12558-s002.jpg]

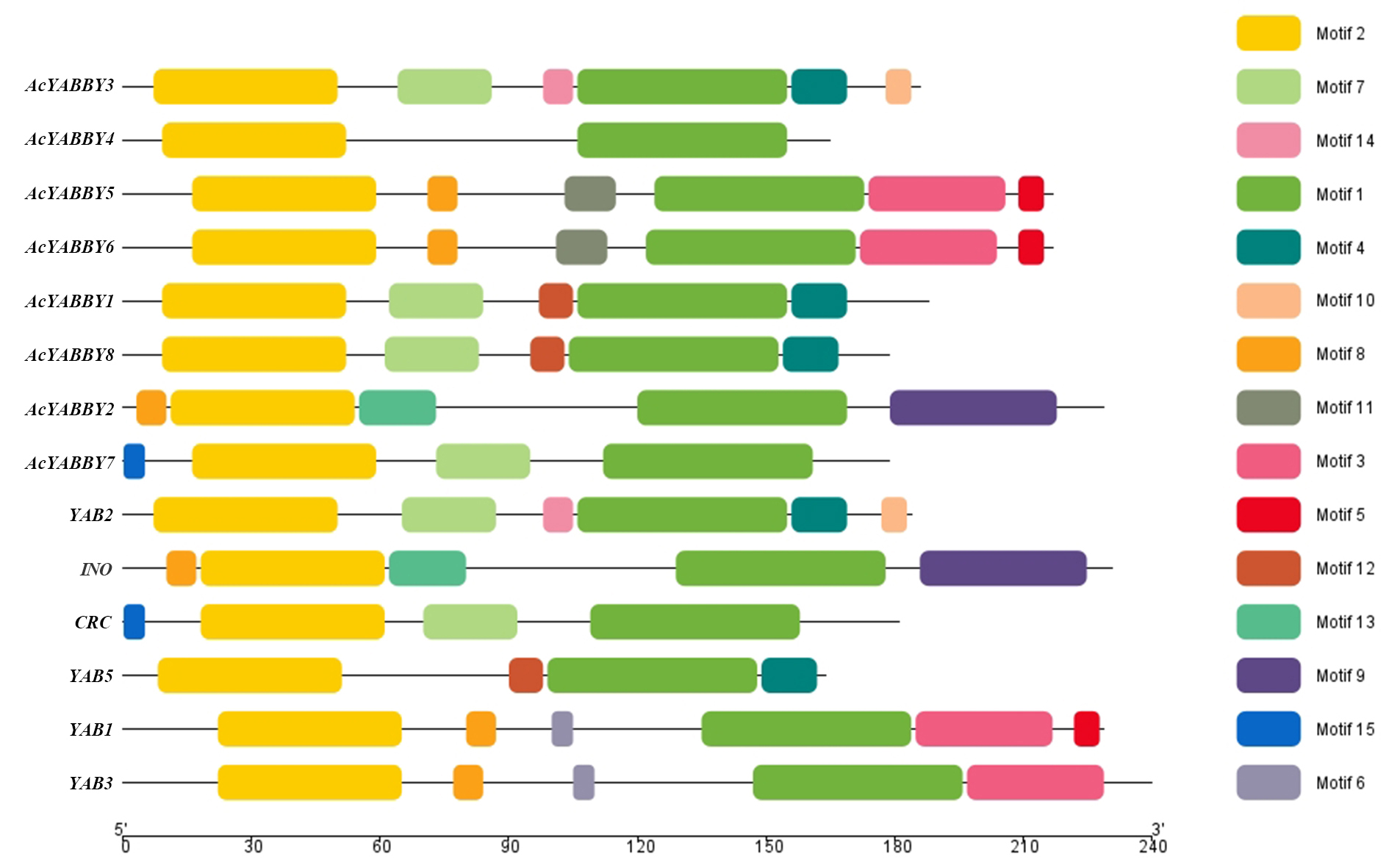

Supplement: Supplemental Information 3 — Grey lines represent the non-conserved sequences, and four conserved motifs are indicated by different colors with numbered boxes. [file peerj-10-12558-s003.jpg]

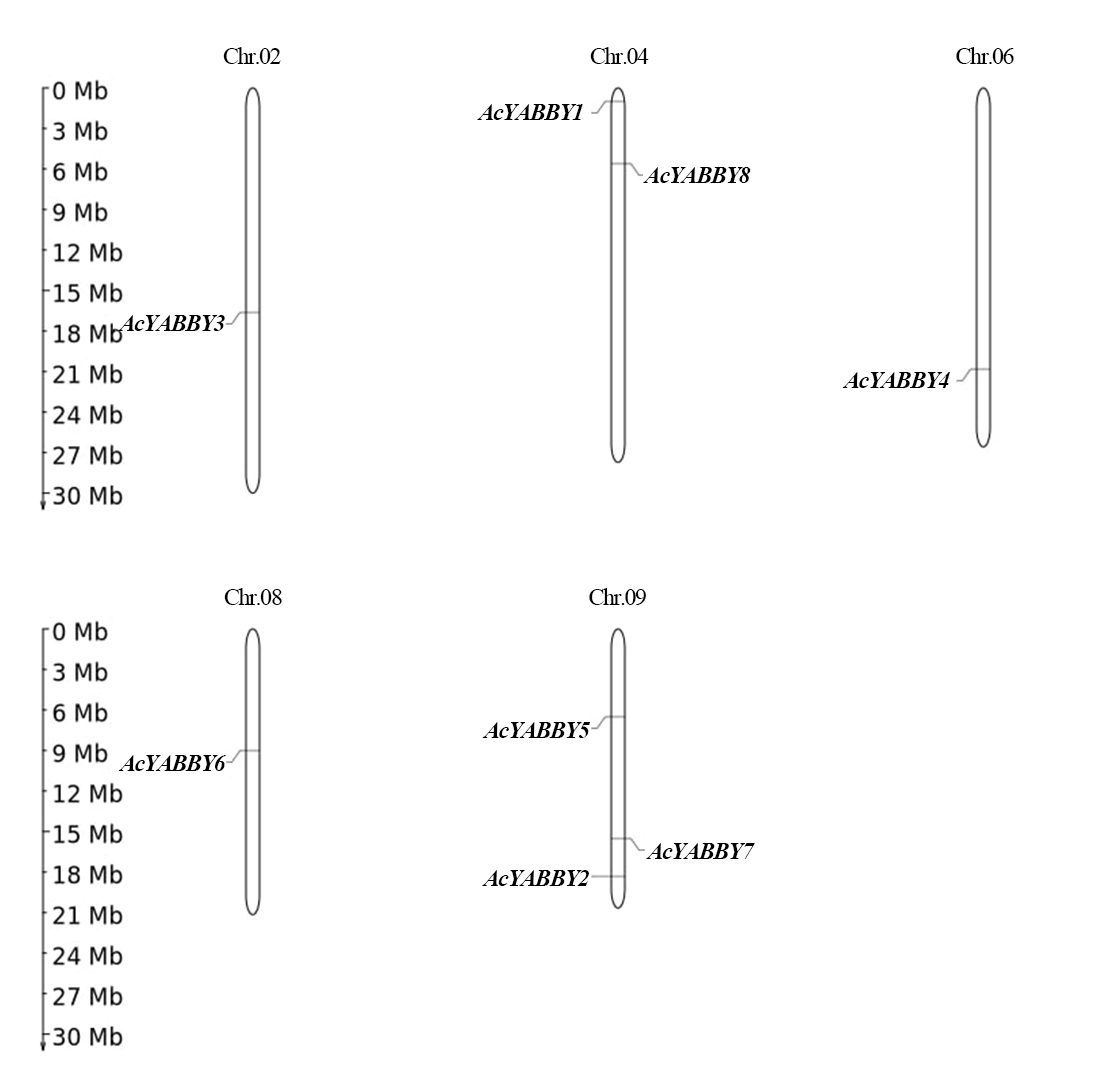

Supplement: Supplemental Information 4 — A total of five chromosomes of star fruit were labeled with their names, Chr.02, Chr.04, Chr.06, Chr.08, and Chr.09, which are indicated at the top of each bar. The position of AcYABBYs on the chromosome was drawn by online software MG2C (http://mg2c.iask.in/mg2c_v2.0/) based on GFF file. [file peerj-10-12558-s004.jpg]

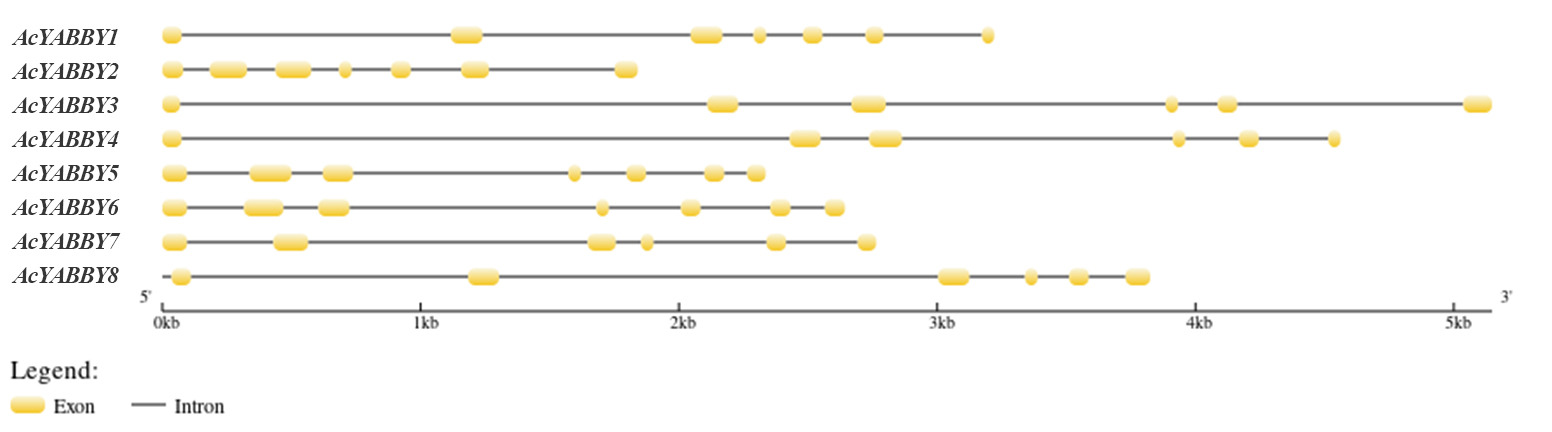

Supplement: Supplemental Information 5 — Exons and introns are represented by yellow rectangle and black lines, respectively. The lengths of exons and introns for each AcYABBY gene are shown proportionally. [file peerj-10-12558-s005.jpg]

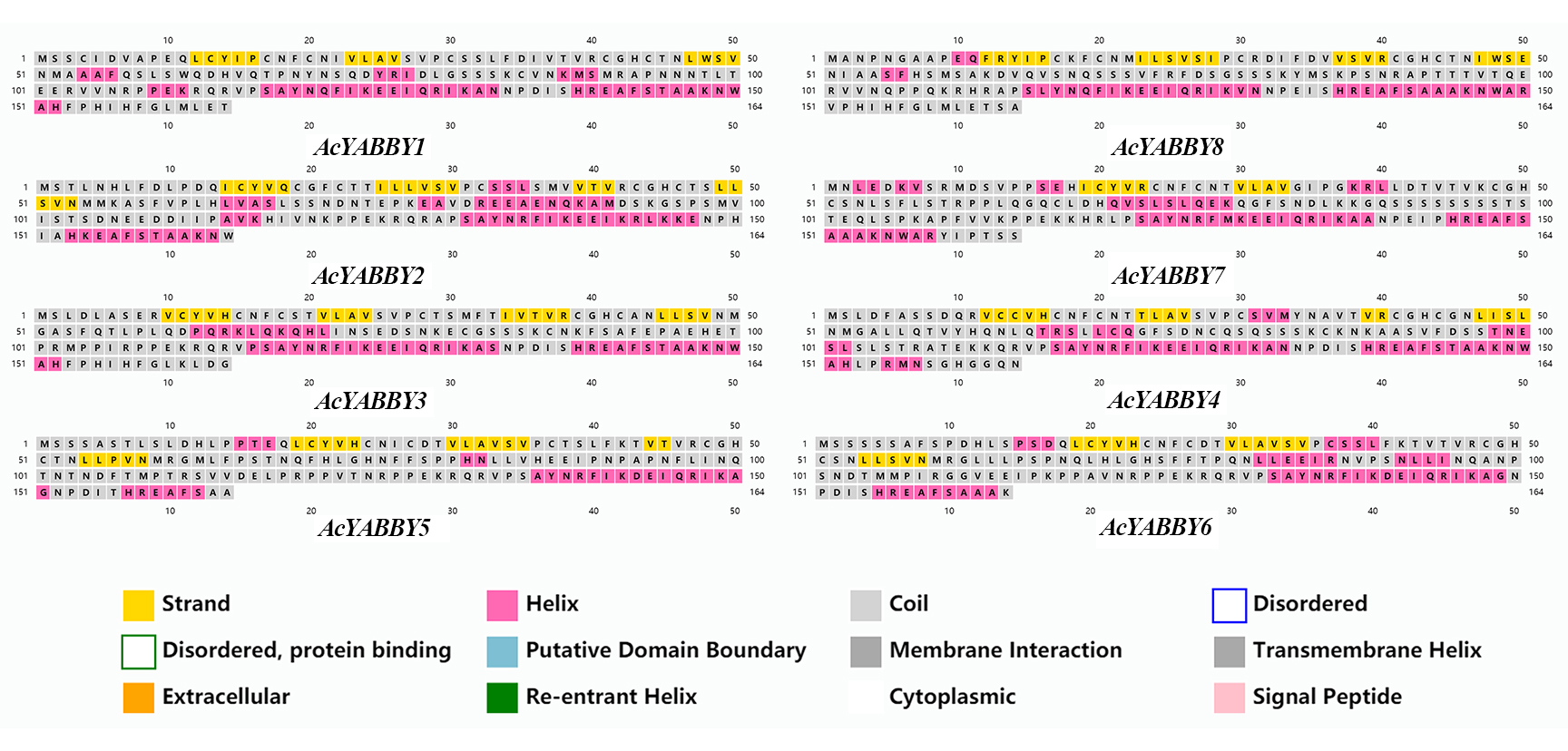

Supplement: Supplemental Information 6 — Different color blocks represent different secondary structures. [file peerj-10-12558-s006.jpg]

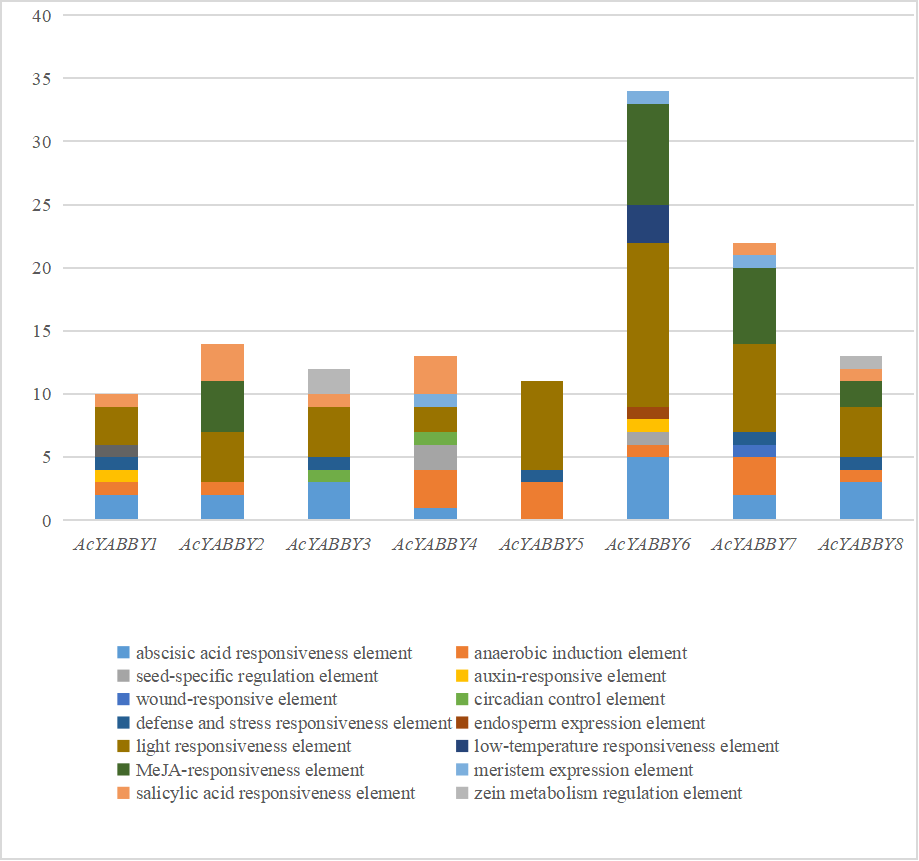

Supplement: Supplemental Information 7 — Note: The numbers of different cis-elements are presented in the form of bar graphs and similar cis-elements are exhibited with the same colors. [file peerj-10-12558-s007.png]
