# Supplementary material for: Genome-wide identification and expression profile of YABBY genes in Averrhoa carambola
Source: PeerJ. 2022 Jan 4;10:e12558. doi: 10.7717/peerj.12558 (PMC8740515; doi:10.7717/peerj.12558)
Supplement: Supplemental Information 10 [file peerj-10-12558-s010.docx]

**Genome-wide identification and expression profile of the YABBY genes in *Averrhoa carambola***

Cheng Ru Li^1^, Na Dong^1^, Li Ming Shen^1^, Meng Lu^1^, Jun Wen Zhai^1^, Ya Mei Zhao^1^, Lei Chen^1^, Zhi Ting Wan^1^, Zhong Jian Liu^1^, Hui Ren^2*^ & Sha Sha Wu^1*^

1. Fujian Ornamental Plant Germplasm Resources Innovation & Engineering Application Research Center, Key Laboratory of National Forestry and Grassland Administration for Orchid Conservation and Utilization at College of Landscape Architecture, Fuzhou 350002, China

2. Horticulture Research Institute, Guangxi Academy of Agricultural Sciences, Nanning 530007, China

**^*^Corresponding authors:**

Hui Ren

Tel: +86-13100510751; E-mail: renhui0988@163.com

Shasha Wu

Tel: +86-15280430239; E-mail: shashawu1984@126.com

Supporting Materials

**Figure S1.** Sample preparations. A: flower bracts; B: inflorescences; C and D: anatomical and front views of the fruits, respectively. (Scale bar: C and D, 2 cm)

**Figure S2.** Domain of YABBY genes in star fruit.

**Figure S3.** Conserved motifs of YABBY genes in *A. thaliana* and star fruit were predicted by MEME. Grey lines represent the non-conserved sequences, and four conserved motifs are indicated by different colors with numbered boxes.

**Figure S4.** Chromosomal locations of *AcYABBYs*. A total of five chromosomes of star fruit were labeled with their names, Chr.02, Chr.04, Chr.06, Chr.08, and Chr.09, which are indicated at the top of each bar. The position of *AcYABBYs* on the chromosome was drawn by online software MG2C (<http://mg2c.iask.in/mg2c_v2.0/>) based on GFF file.

**Figure S5.** Gene structure of the *AcYABBYs* in star fruit. Exons and introns are represented by yellow rectangle and black lines, respectively. The lengths of exons and introns for each *AcYABBY* gene are shown proportionally.

**Figure S6.** The analysis of YABBY protein secondary structures. Different color blocks represent different secondary structures.

**Figure S7.** Cis-acting elements of *AcYABBYs* in promoter regions. *Note:* The numbers of different cis-elements are presented in the form of bar graphs and similar cis-elements are exhibited with the same colors.

**Table S1.** The primers of *AcYABBYs* and reference genes

**Table S2.** The basic information of YABBY family members in *Ave. carambola*.

**Table S3.** Subcellular localization prediction using three protein subcellular location prediction tools

**Table S4.** *YABBY* genes in *A. thaliana*, *O. sativa*, *S. lycopersicum*, *V. vinifera*, *Z. mays*, and *Ave. carambola*.

**Table S5.** Selective pressure analysis of *AcYABBYs*.

**Data S1.** The sequence of eight *AcYABBYs*.

**
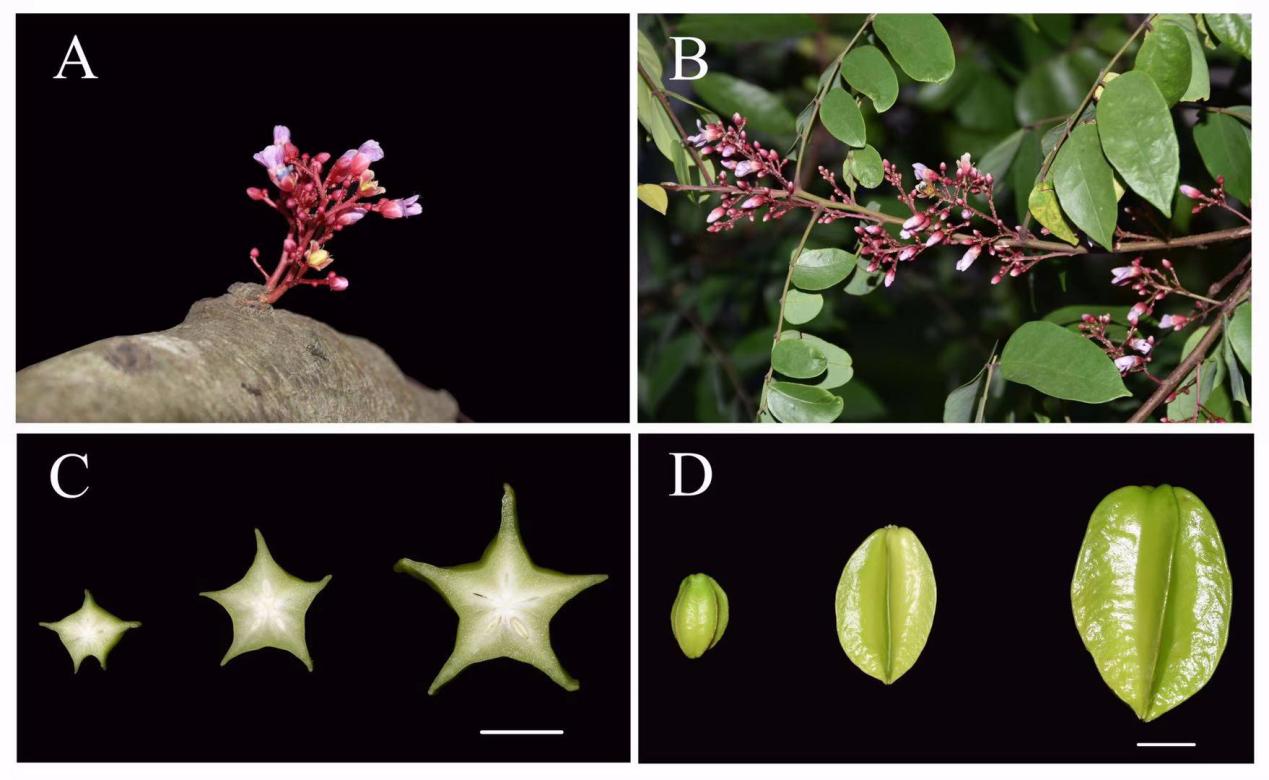
**

**Figure S1.** Sample preparations. A: flower bracts; B: inflorescences; C: anatomical view of the fruits 20, 40, 60 days after pollination, respectively; D: . (Scale bar: C and D, 2 cm).


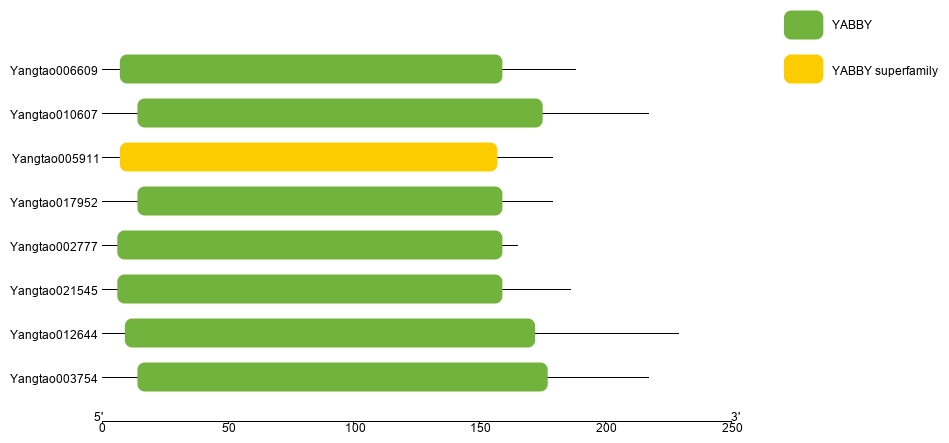


**Figure S2.** Domain of YABBY genes in star fruit.


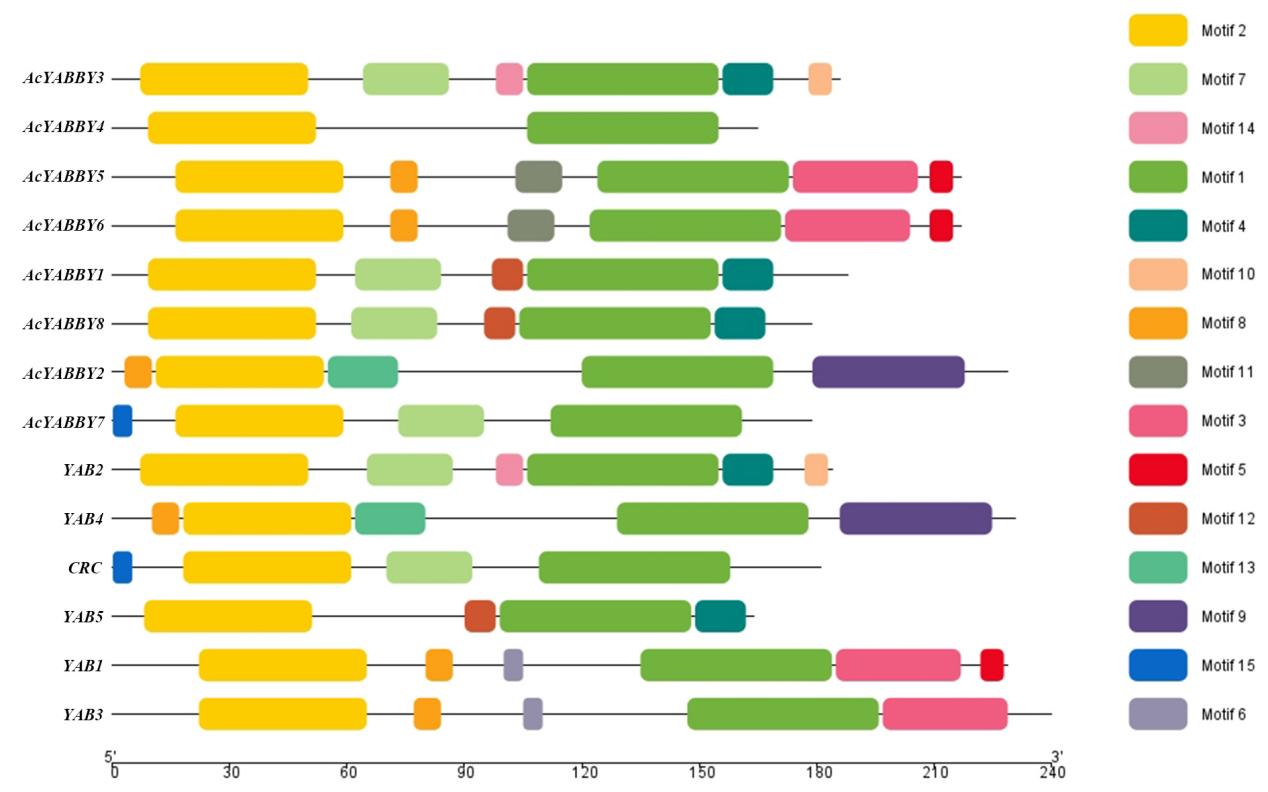


**Figure S3.** Conserved motifs of YABBY genes in *A. thaliana* and star fruit were predicted by MEME. Grey lines represent the non-conserved sequences, and four conserved motifs are indicated by different colors with numbered boxes.

**
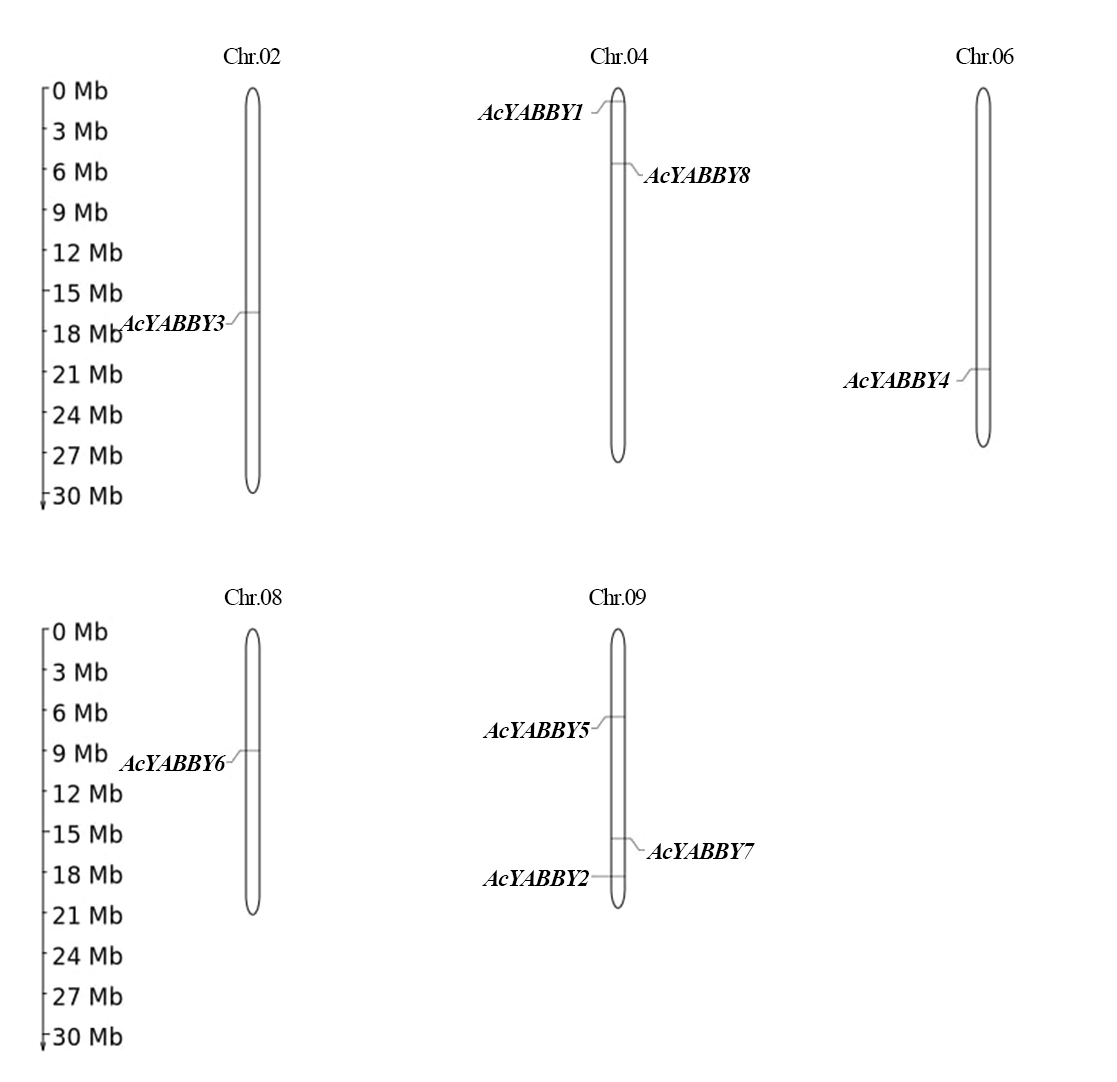
**

**Figure S4.** Chromosomal locations of *AcYABBYs*. A total of five chromosomes of star fruit were labeled with their names, Chr.02, Chr.04, Chr.06, Chr.08, and Chr.09, which are indicated at the top of each bar. The position of *AcYABBYs* on the chromosome was drawn by online software MG2C (<http://mg2c.iask.in/mg2c_v2.0/>) based on GFF file.


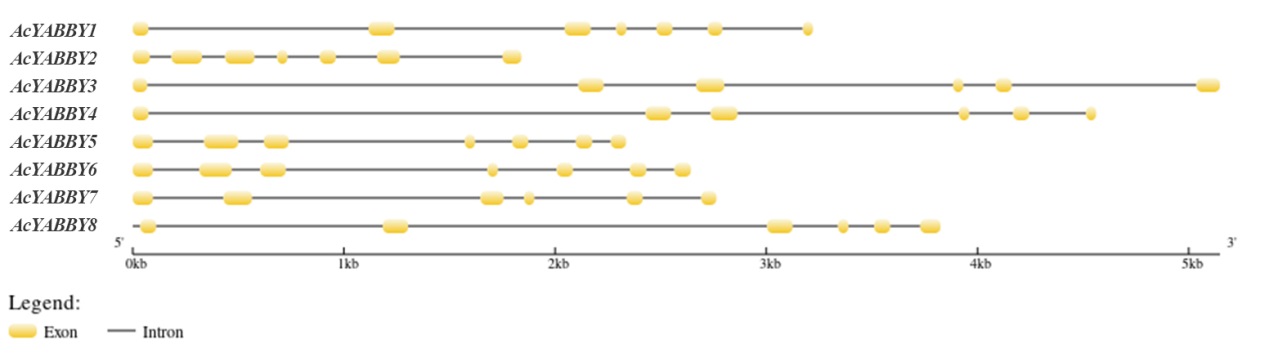


**Figure S5.** Gene structure of the *AcYABBYs* in star fruit. Exons and introns are represented by yellow rectangle and black lines, respectively. The lengths of exons and introns for each *AcYABBY* gene are shown proportionally.


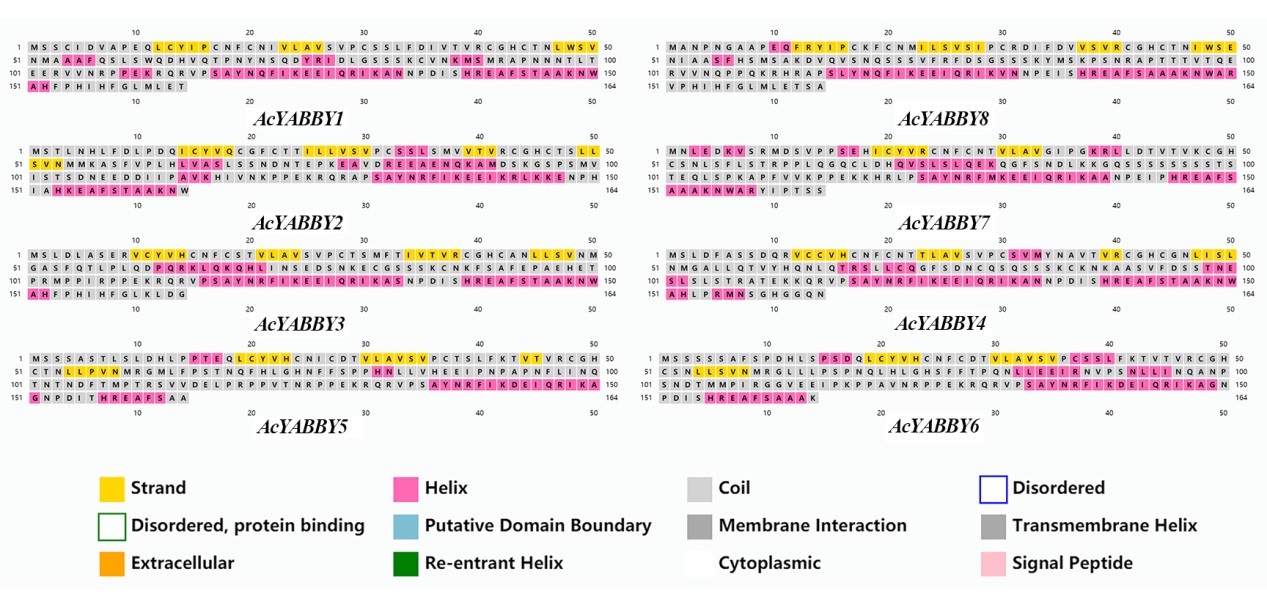


**Figure S6.** The analysis of YABBY protein secondary structures. Different color blocks represent different secondary structures.


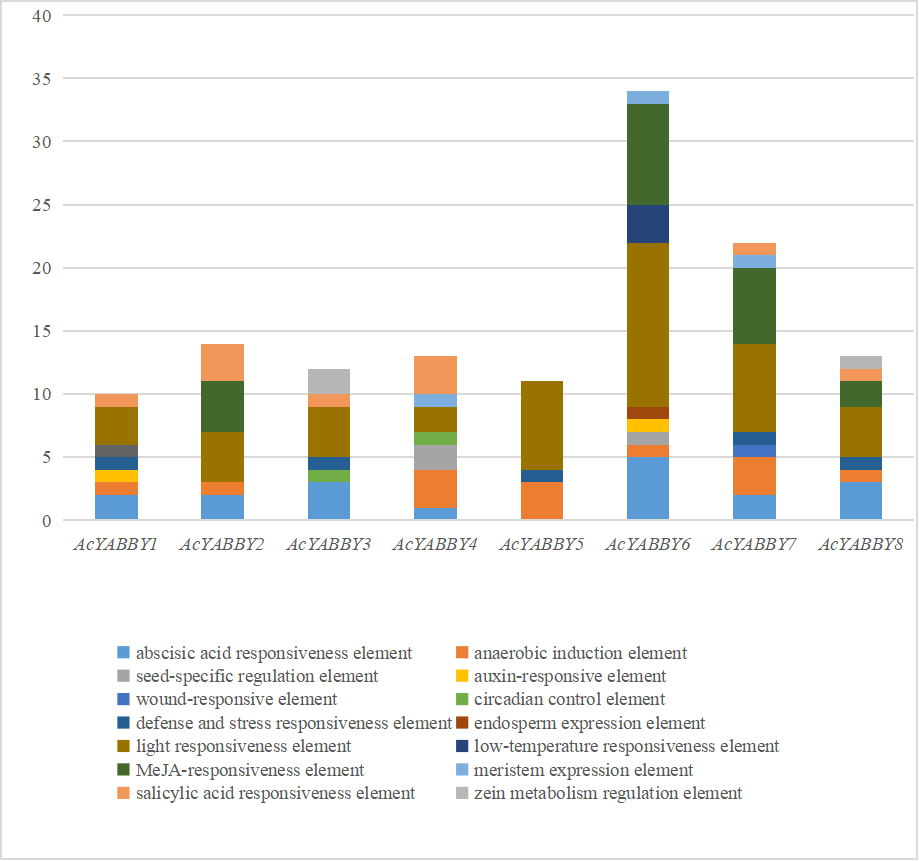


**Figure S7.** Cis-acting elements of *AcYABBYs* in promoter regions. *Note:* The numbers of different cis-elements are presented in the form of bar graphs and similar cis-elements are exhibited with the same colors.

**Table S1.** The primers of *AcYABBYs* and reference genes

| Genes | （5′–3′）Forward primer | （5′–3′）Reverse primer |
| --- | --- | --- |
| *AcYABBY1* | CAACTCTGCTACATCCCTTGC | GATCTTGCCATGAAAGTGACTG |
| *AcYABBY2* | ACGTGCAATGTGGTTTCTG | TGACTGAGAGAAGACTTGTGCAG |
| *AcYABBY3* | GAGAGAGTTTGTTATGTTCACTGCA | GAGGAAGTGTTTGAAATGAAGCTC |
| *AcYABBY4* | CTCGACTTTGCTTCTTCTGATC | CTTTGTTGCAGACTGTCTATCATC |
| *AcYABBY5* | CTCCTCAGCTTCTACCTTGTCTTT | GCATATTCACAGGCAGGAGAT |
| *AcYABBY6* | TCATCTGCTTTTTCACCGG | GCAGTCCACGCATGTTAACT |
| *AcYABBY7* | CACCATCCGAGCACATCT | AGTGGATCTGGTGCTGAGAAA |
| *AcYABBY8* | GCACCTGAGCAATTTCGC | GTTGGAGACCTGGACATCC |
| *ACTB* | CAGTGTCTGGATTGGAGGA | ATCTGTTGGAAGGTGCTGA |

**Table S2.** The basic information of YABBY family members in *Ave. carambola*.

| Nomenclature | Gene ID | Subfamily | Acession ID | Length of protein | MW (Da) | PI | Subcellular location |
| --- | --- | --- | --- | --- | --- | --- | --- |
| Yangtao006609 | *AcYABBY1* | YAB5 | MW014334 | 194 | 21287.25 | 8.13 | Nuclear |
| Yangtao012644 | *AcYABBY2* | INO | MW014336 | 235 | 25197.26 | 5.22 | Nuclear |
| Yangtao021545 | *AcYABBY3* | YAB2 | MW014333 | 192 | 20881.77 | 8.81 | Nuclear |
| Yangtao002777 | *AcYABBY4* | YAB2 | MW014337 | 169 | 18053.34 | 9.00 | Nuclear |
| Yangtao003754 | *AcYABBY5* | FIL/YAB3 | MW014335 | 223 | 24246.62 | 7.72 | Nuclear |
| Yangtao010607 | *AcYABBY6* | FIL/YAB3 | MW014332 | 223 | 23901.23 | 8.25 | Nuclear |
| Yangtao017952 | *AcYABBY7* | CRC | MW014338 | 183 | 19689.35 | 9.56 | Nuclear |
| Yangtao005911 | *AcYABBY8* | YAB5 | MW014339 | 183 | 19886.57 | 9.37 | Nuclear |

**Table S3.** Subcellular localization prediction using three protein subcellular location prediction tools

| Proteins | LocTree3 (confidence%) | PSORT (confidence%) | Softberry (score) |
| --- | --- | --- | --- |
| *AcYABBY3* | 89%: nucleus | 69.6 %: nuclear | Nuclear: 9.1 |
| *AcYABBY4* | 91%: nucleus | 78.3 %: nuclear | Nuclear: 9 |
| *AcYABBY5* | 87%: nucleus | 69.6 %: nuclear | Nuclear: 9.1 |
| *AcYABBY6* | 89%: nucleus | 73.9 %: nuclear | Nuclear: 9.1 |
| *AcYABBY1* | 94%: nucleus | 69.6 %: nuclear | Nuclear: 9 |
| *AcYABBY8* | 92%: nucleus | 78.3 %: nuclear | Nuclear: 9 |
| *AcYABBY2* | 88%: nucleus | 82.6 %: nuclear | Nuclear: 8.8 |
| *AcYABBY7* | 89%: nucleus | 82.6 %: nuclear | Nuclear: 8.9 |

**Table S4.** *YABBY* genes in *A. thaliana*, *O. sativa*, *S. lycopersicum*, *V. vinifera*, *Z. mays*, and *Ave. carambola*.

| Subfamilies | *A. thaliana* | *O. sativa* | *S. lycopersicum* | *V. vinifera* | *Z. mays* | *Ave. carambola* |
| --- | --- | --- | --- | --- | --- | --- |
| YAB3/FIL | 1 | 3 | 2 | 2 | 5 | 2 |
| YAB2 | 1 | 3 | 2 | 2 | 5 | 2 |
| YAB5 | 1 | 0 | 2 | 1 | 0 | 2 |
| INO | 1 | 1 | 1 | 1 | 1 | 1 |
| CRC | 1 | 1 | 2 | 1 | 2 | 1 |
| Total | 6 | 8 | 9 | 7 | 13 | 8 |

**Table S5.** Selective pressure analysis of *AcYABBYs*.

| Gene pairs | Ka | Ks | Ka/Ks |
| --- | --- | --- | --- |
| *AcYABBY1*-*AcYABBY2* | 0.480962 | 2.66364 | 0.180566 |
| *AcYABBY1*-*AcYABBY7* | 0.479474 | 2.65874 | 0.180338 |
| *AcYABBY1*-*AcYABBY8* | 0.241087 | 0.877205 | 0.274836 |
| *AcYABBY2*-*AcYABBY7* | 0.653473 | 2.08623 | 0.313231 |
| *AcYABBY3*-*AcYABBY1* | 0.253378 | 3.51706 | 0.072043 |
| *AcYABBY3*-*AcYABBY2* | 0.43698 | 2.87199 | 0.152152 |
| *AcYABBY3*-*AcYABBY4* | 0.28933 | 2.13382 | 0.135593 |
| *AcYABBY3*-*AcYABBY5* | 0.339389 | 3.17847 | 0.106777 |
| *AcYABBY3*-*AcYABBY6* | 0.338746 | 3.2288 | 0.104914 |
| *AcYABBY3*-*AcYABBY7* | 0.479383 | 2.58086 | 0.185745 |
| *AcYABBY3*-*AcYABBY8* | 0.422429 | 2.82667 | 0.149444 |
| *AcYABBY4*-*AcYABBY1* | 0.383274 | 3.1167 | 0.122974 |
| *AcYABBY4*-*AcYABBY2* | 0.498694 | 2.64518 | 0.188529 |
| *AcYABBY4*-*AcYABBY5* | 0.475185 | 2.76021 | 0.172155 |
| *AcYABBY4*-*AcYABBY6* | 0.441681 | 2.87623 | 0.153562 |
| *AcYABBY4*-*AcYABBY7* | 0.54048 | 2.45434 | 0.220214 |
| *AcYABBY4*-*AcYABBY8* | 0.471921 | 2.82097 | 0.16729 |
| *AcYABBY5*-*AcYABBY1* | 0.444926 | 2.91518 | 0.152624 |
| *AcYABBY5*-*AcYABBY2* | 0.591704 | 2.33286 | 0.253639 |
| *AcYABBY5*-*AcYABBY6* | 0.14116 | 1.00591 | 0.140331 |
| *AcYABBY5*-*AcYABBY7* | 0.556525 | 2.34785 | 0.237036 |
| *AcYABBY5*-*AcYABBY8* | 0.56551 | 2.42067 | 0.233617 |
| *AcYABBY6*-*AcYABBY1* | 0.428934 | 2.92835 | 0.146477 |
| *AcYABBY6*-*AcYABBY2* | 0.59031 | 2.29868 | 0.256804 |
| *AcYABBY6*-*AcYABBY7* | 0.54795 | 2.36836 | 0.231362 |
| *AcYABBY6*-*AcYABBY8* | 0.535098 | 2.47645 | 0.216075 |
| *AcYABBY8*-*AcYABBY2* | 0.533001 | 2.49644 | 0.213504 |
| *AcYABBY8*-*AcYABBY7* | 0.554948 | 2.3545 | 0.235696 |

*Note:* Ka indicates the number of [nonsynonymous substitutions](https://en.wikipedia.org/wiki/Nonsynonymous_substitution) per non-synonymous site; Ks indicates the number of [synonymous substitutions](https://en.wikipedia.org/wiki/Synonymous_substitution) per synonymous site. 0< Ka/Ks <1, negative selection; Ka/Ks =1, neutral selection; Ka/Ks >1, positive selection.

**Data S1.** The sequences of eight *AcYABBYs*.

>*AcYABBY3*

MSLDLASERVCYVHCNFCSTVLAVSVPCTSMFTIVTVRCGHCANLLSVNMGASFQTLPLQ

DPQRKLQKQHLINSEDSNKECGSSSKCNKFSAFEPAEHETPRMPPIRPPEKRQRVPSAYN

RFIKEEIQRIKASNPDISHREAFSTAAKNWAHFPHIHFGLKLDGNKQARMEQALEEGTQK

SNGFY*

>*AcYABBY4*

MSLDFASSDQRVCCVHCNFCNTTLAVSVPCSVMYNAVTVRCGHCGNLISLNMGALLQTVY

HQNLQTRSLLCQGFSDNCQSQSSSKCKNKAASVFDSSTNESLSLSTRATEKKQRVPSAYN

RFIKEEIQRIKANNPDISHREAFSTAAKNWAHLPRMNSGHGGQN*

>*AcYABBY5*

MSSSASTLSLDHLPPTEQLCYVHCNICDTVLAVSVPCTSLFKTVTVRCGHCTNLLPVNMR

GMLFPSTNQFHLGHNFFSPPHNLLVHEEIPNPAPNFLINQTNTNDFTMPTRSVVDELPRP

PVTNRPPEKRQRVPSAYNRFIKDEIQRIKAGNPDITHREAFSAAAKNWAHFPHIHFGLMP

DQTTVKKTNMRPQEGDDVLMKENGYFASANVGVSPY*

>*AcYABBY6*

MSSSSSAFSPDHLSPSDQLCYVHCNFCDTVLAVSVPCSSLFKTVTVRCGHCSNLLSVNMR

GLLLPSPNQLHLGHSFFTPQNLLEEIRNVPSNLLINQANPSNDTMMPIRGGVEEIPKPPA

VNRPPEKRQRVPSAYNRFIKDEIQRIKAGNPDISHREAFSAAAKNWAHFPHIHFGLLPDH

QPVKKANVRQQEGEDVLMKDGFFTAPPATNVGVTPY*

>*AcYABBY1*

MSSCIDVAPEQLCYIPCNFCNIVLAVSVPCSSLFDIVTVRCGHCTNLWSVNMAAAFQSLS

WQDHVQTPNYNSQDYRIDLGSSSKCVNKMSMRAPNNNTLTEERVVNRPPEKRQRVPSAYN

QFIKEEIQRIKANNPDISHREAFSTAAKNWAHFPHIHFGLMLETNNQPKMDDGPEKHLMP

RTALLHK*

>*AcYABBY8*

MANPNGAAPEQFRYIPCKFCNMILSVSIPCRDIFDVVSVRCGHCTNIWSENIAASFHSMS

AKDVQVSNQSSSVFRFDSGSSSKYMSKPSNRAPTTTVTQERVVNQPPQKRHRAPSLYNQF

IKEEIQRIKVNNPEISHREAFSAAAKNWARVPHIHFGLMLETSANSTKLDDVSAIAVK*

>*AcYABBY2*

MSTLNHLFDLPDQICYVQCGFCTTILLVSVPCSSLSMVVTVRCGHCTSLLSVNMMKASFV

PLHLVASLSSNDNTEPKEAVDREEAENQKAMDSKGSPSMVISTSDNEEDDIIPAVKHIVN

KPPEKRQRAPSAYNRFIKEEIKRLKKENPHIAHKEAFSTAAKNWAQNPPMQFNRDEESCG

QEVEEGEGEATWNPNAVEGVDIQGNGFRERKSPEGLDTGSKNNFSTVA*

>*AcYABBY7*

MNLEDKVSRMDSVPPSEHICYVRCNFCNTVLAVGIPGKRLLDTVTVKCGHCSNLSFLSTR

PPLQGQCLDHQVSLSLQEKQGFSNDLKKGQSSSSSSSSTSTEQLSPKAPFVVKPPEKKHR

LPSAYNRFMKEEIQRIKAANPEIPHREAFSAAAKNWARYIPTSSNGSVSRTSNNVRNI*
